# Narrow-Bandpass One-Step Leapfrog Hybrid Implicit-Explicit Algorithm with Convolutional Boundary Condition for Its Applications in Sensors

**DOI:** 10.3390/s22124445

**Published:** 2022-06-12

**Authors:** Yangjing Wang, Yongjun Xie, Haolin Jiang, Peiyu Wu

**Affiliations:** 1 School of Electronic and Information Engineering, Beihang University, Beijing 100191, China; piratewyj@163.com (Y.W.); yjxie@buaa.edu.cn (Y.X.); 2 Beijing Key Laboratory of Microwave Sensing and Security Applications, Beihang University, Beijing 100191, China; 3 Shenzhen Institute of Beihang University, Shenzhen 518000, China; 4 Xi’an Institute of Space Radio Technology, Xi’an 710100, China; 5 School of Electronic and Information Engineering, Nanjing University of Information Science and Technology, Nanjing 210096, China; haolinjiang.cem@gmail.com

**Keywords:** finite-difference time-domain (FDTD), hybrid implicit-explicit (HIE), convolutional perfectly matched layer (CPML), complex envelope (CE), bandpass sensors and components

## Abstract

A large number of sensors work in the narrow bandpass circumstance. Meanwhile, some of them hold fine details merely along one and two dimensions. In order to efficiently simulate these sensors and devices, the one-step leapfrog hybrid implicit-explicit (HIE) algorithm with the complex envelope (CE) method and absorbing boundary condition is proposed in the narrow bandpass circumstance. To be more precise, absorbing boundary condition is implemented by the higher order convolutional perfectly matched layer (CPML) formulation to further enhance the absorption during the entire simulation. Numerical examples and their experiments are carried out to further illustrate the effectiveness of the proposed algorithm. The results show considerable agreement with the experiment and theory resolution. The relationship between the time step and mesh size can break the Courant–Friedrichs–Levy condition which indicates the physical size/selection mesh size. Such a condition indicates that the proposed algorithm behaviors are considerably accurate due to the rational choice in discretized mesh. It also shows decrement in simulation duration and memory consumption compared with the other algorithms. In addition, absorption performance can be improved by employing the proposed higher order CPML algorithm during the whole simulation.

## 1. Introduction

With the development of the electronic industry, sensors have become more important than ever before. Large numbers of sensors work in narrow bandpass circumstances or individual frequency [1,2]. Although the finite-difference time-domain (FDTD) algorithm has been extensively employed in many applications, sensor simulation by the FDTD algorithm is severely limited by the inefficient bandpass calculation. Such conditions also restrict the development of the FDTD algorithm [3]. The reason is that the FDTD algorithm is based on the lowpass-sampling-theorem which can achieve considerable performance merely in broadband simulation [4]. The time step becomes small, resulting in an unacceptable simulation duration in bandpass problems. In order to efficiently simulate bandpass problems, investigation of the alternative FDTD algorithm has become urgent. Complex envelope (CE) method which is based on the bandpass-sampling-theorem shows considerable potential in bandpass problems [5]. In the CE method, the mesh size can be chosen according to the bandwidth rather than the maximum frequency, resulting in significantl improvement, both from the aspect of efficiency and accuracy. The CE method was firstly employed into the conventional FDTD algorithm [6]. Then, it was introduced into the unconditionally stable algorithms [7,8,9,10]. Through results, it can be concluded that these algorithms can receive better behavior compared with the lowpass-sampling-theorem based algorithms in bandpass simulation.

With the development of sensors, structures have become more complex than ever before. For the fine details simulation, mesh size must be fine enough to satisfy the calculation accuracy. Due to the existence of the Courant–Friedrichs–Levy (CFL) condition, the relationship between time step and mesh size is established [11]. Thus, time step must also be fine enough to maintain the stability of the algorithm. Such conditions also result in an extremely long simulation duration. In order to alleviate such conditions, unconditionally stable algorithms are proposed including the alternating direction implicit (ADI), locally one-dimension (LOD), split-step (SS) procedures and others [12,13,14,15]. It has been testified that the unconditionally stable algorithms are merely efficient in fine details with all directions. When simulating fine details along one and two dimensions, more than six matrices should be calculated at each time step resulting in increments of memory consumption and simulation duration. Although these algorithms can overcome the CFL condition, the effectiveness of the algorithm decreases significantly. In order to alleviate such conditions, a hybrid implicit-explicit (HIE) procedure is proposed [16]. Through explicitly updating components in a single direction and implicitly updating the others, the HIE procedure can obtain considerable performance in low-dimensions [17,18]. However, the HIE procedure is still a split-field updating procedure which increases the memory and simulation duration [19]. To alleviate such conditions, the one-step leapfrog scheme is employed into the HIE procedure [20].

Because the computer cannot solve infinite computational domains, an adequate boundary condition must be employed at the boundaries [11]. To simulate infinite computational domain in finite space, perfectly matched layer (PML) is regarded as one of the most powerful and efficient absorbing boundary conditions [21]. The original PML formulation is a split-field scheme which shows limitations in absorption and resources [22]. Unsplit-field formulation is proposed to alleviate such conditions including convolutional PML (CPML), stretched coordinate PML (SC-PML) and complex-frequency-shifted PML (CFS-PML) schemes [23,24,25]. Although they hold advantages in reducing late-time reflections and absorbing low-frequency evanescent waves, reflection at the low frequency still needs to be enhanced [26]. Higher order PML formulation is proposed to not only enhance the absorption in the time domain but also absorb low-frequency propagation waves in the frequency domain [27,28]. The original higher order PML formulation must introduce six auxiliary variables during calculation which occupies large number of computational resources [29]. To alleviate such conditions, the higher order PML formulation with four auxiliary variables is proposed [30]. In addition, existing higher order formulation is mainly based on the CFS-PML scheme which shows limitation in complex media. The higher order CPML is proposed to overcome these drawbacks [31,32]. It has been testified that compared with the other schemes, it can significantly decrease the computational complexity with considerable absorption in complex structures and media.

In order to simulate complex sensors and components whose fine details merely exist along one and two dimensions, several algorithms have been proposed which still have their own limitations. The original HIE procedures with PML (NPML) and CFS-PML are proposed in two dimensions which cannot be employed into the practical employment [33,34]. Recently, the HIE procedure and its leapfrog scheme with higher order PML are proposed in three dimensions [35,36,37]. However, they can merely be employed in vacuum and magnetized plasma. The formulation for dielectric material still needs to be investigated. From another aspect, all of the existing absorbing boundary conditions for the HIE procedure and leapfrog scheme are based on the NPML and CFS-PML formulation [35,36,37,38,39]. These algorithms must be modified according to the different computational domains due to the media-dependent formulation. Computation complexity increases significantly due to the employment of higher order formulation. The reduction in computation complexity also needs to be further investigated.

In order to efficiently simulate sensors with fine details in narrow bandpass circumstances, the one-step leapfrog HIE procedure is proposed with the CE method and higher order CPML formulation. The proposed algorithm takes advantages of leapfrog HIE procedure, CE method and higher order CPML formulation, in terms of the calculating of fine details along one and two dimensions, simulating bandpass problems and enhancing absorption. Numerical examples and experiments are carried out for the illustration of effectiveness. It can be concluded that results simulated by employing the proposed algorithm show considerable agreement with the experiments and theory resolution. Compared to the other algorithms, the proposed algorithm also shows significant improvement in accuracy, efficiency and absorption.

## 2. Formulation

Because massive components exist in Maxwell’s equations, assuming the fine details merely locate along x-direction, $E_{y}$ and $H_{z}$ components are selected as examples for demonstration. The other components can be obtained by employing the similar approach. The Maxwell’s equations inside the dielectric material can be given as follows:

(1a) $$j\omega \varepsilon_{0}E_{y}+\sigma E_{y}=S_{z}^{-1}\partial_{z}H_{x}-S_{x}^{-1}\partial_{x}H_{z}$$

(1b) $$-j\omega \mu_{0}H_{y}=S_{z}^{-1}\partial_{z}E_{x}-S_{x}^{-1}\partial_{x}E_{z}$$

where $S_{\eta },\;\eta =x,y,z$ is the stretched coordinate variable side PML regions with higher order formulation which can be defined as:

(1c) $$S_{\eta }=\left(\kappa_{\eta 1}+\frac{\sigma_{\eta 1}}{\alpha_{\eta 1}+j\omega \varepsilon_{0}}\right)\left(\kappa_{\eta 2}+\frac{\sigma_{\eta 2}}{\alpha_{\eta 2}+j\omega \varepsilon_{0}}\right)$$

where $\sigma_{\eta n},\;n=1,2$ and $\alpha_{\eta n}$ are supposed to be positive real, $\kappa_{\eta n}$ is supposed to be real. According to the CPML formulation, by employing the partial fraction expansion to the stretched coordinate variables, results can be converted by employing the inverse Laplace transform, one obtains:

(2) $${\bar{S}}_{\eta }\left(t\right)=\left(\frac{\delta \left(t\right)}{\kappa_{\eta 1}}+\zeta_{\eta 1}\left(t\right)\right)\left(\frac{\delta \left(t\right)}{\kappa_{\eta 2}}+\zeta_{\eta 2}\left(t\right)\right)$$

where the coefficients can be given as $\zeta_{\eta n}=-r_{\eta n}e^{-v_{\eta n}t}u(t)$, $r_{\eta n}=-\sigma_{\eta n}/\left(\kappa_{\eta n}^{2}\varepsilon_{0}\right)$, $v_{\eta n}=\alpha_{\eta n}/\varepsilon_{0}+\sigma_{\eta n}/\left(\kappa_{\eta n}\varepsilon_{0}\right)$. In addition, δ(t) and u(t) represent the unit speed function and unit impulse function. It can be observed that the original Maxwell’s equations obey the lowpass sampling-theorem which shows performance decrement with the bandpass simulation. In order to alleviate such conditions, the CE method is introduced into the Maxwell’s equations [5]. According to the CE method, the original signal can be given as the complex envelope form, one obtains:

(3) Φ=Re{Φ^ejϖt}

where Re{⋅} represents the real number in the complex signal, Φ and $\hat{\Phi }$ represent the original signal and complex envelope signal, ϖ is the center frequency of the modulated signal. According to the CE method and CPML formulation, Maxwell’s equations can be given as:

(4a) $$\left(j\varpi +\partial t\right){\hat{E}}_{y}+\sigma {\hat{E}}_{y}={\bar{S}}_{z}\left(t\right)*\partial_{z}{\hat{H}}_{x}-{\bar{S}}_{x}\left(t\right)*\partial_{x}{\hat{H}}_{z}$$

(4b) $$\left(j\varpi +\partial_{t}\right){\hat{H}}_{z}={\bar{S}}_{x}\left(t\right)*\partial_{x}{\hat{E}}_{z}-{\bar{S}}_{z}\left(t\right)*\partial_{z}{\hat{E}}_{x}$$

In order to update the equations, Equation (2) is substituted into Equation (4a,b), one obtains:

(5a) $$\left(j\varpi +\partial t\right){\hat{E}}_{y}+\sigma {\hat{E}}_{y}=\;{\hat{F}}_{yz12}+\kappa_{z1}^{-1}{\hat{F}}_{yz1}+\kappa_{z2}^{-1}{\hat{F}}_{yz2}+\kappa_{z1}^{-1}\kappa_{z2}^{-1}\partial_{z}{\hat{H}}_{x}^{}-{\hat{F}}_{yx12}-\kappa_{x1}^{-1}{\hat{F}}_{yx1}-\kappa_{x2}^{-1}{\hat{F}}_{yx2}+\kappa_{x1}^{-1}\kappa_{x2}^{-1}\partial_{x}{\hat{H}}_{z}^{}$$

(5b) $$\left(j\varpi +\partial_{t}\right){\hat{H}}_{z}=\;{\hat{G}}_{zx12}+\kappa_{x1}^{-1}{\hat{G}}_{zx1}+\kappa_{x2}^{-1}{\hat{G}}_{zx2}+\kappa_{x1}^{-1}\kappa_{x2}^{-1}\partial_{x}{\hat{E}}_{y}^{}-{\hat{G}}_{zy12}-\kappa_{y1}^{-1}{\hat{G}}_{zy1}-\kappa_{y2}^{-1}{\hat{G}}_{zy2}-\kappa_{y1}^{-1}\kappa_{y2}^{-1}\partial_{y}{\hat{E}}_{x}^{}$$

where F and G are the auxiliary variables. By rewriting the equations into the discretized domain for updating, the results can be given according to the sub-step procedure as:

*The First Time Step*:

(6a) $${\hat{E}}_{y}^{n+1/2}-p_{3x}\delta_{x}{\hat{H}}_{z}^{n+1/2}+p_{3z}\delta_{z}{\hat{H}}_{x}^{n+1/2}=\;k_{1}c_{1}{\hat{E}}_{y}^{n}+p_{4z}{\hat{F}}_{yz12}^{n}+p_{1z}{\hat{F}}_{yz1}^{n}+p_{2z}{\hat{F}}_{yz2}^{n}-p_{4x}{\hat{F}}_{yx12}^{n}-p_{1x}{\hat{F}}_{yx1}^{n}-p_{2x}{\hat{F}}_{yx2}^{n}$$

(6b) $${\hat{H}}_{z}^{n+1/2}-p_{3x}\delta_{x}{\hat{E}}_{y}^{n+1/2}=\;c_{1}{\hat{H}}_{z}^{n}+p_{4x}{\hat{G}}_{zx12}^{n}+p_{1x}{\hat{G}}_{zx1}^{n}+p_{2x}{\hat{G}}_{zx2}^{n}-p_{4y}{\hat{G}}_{zy12}^{n}-p_{1y}{\hat{G}}_{zy1}^{n}-p_{2y}{\hat{G}}_{zy2}^{n}-p_{3y}\delta_{y}{\hat{E}}_{x}^{n}$$

*The Second Time Step*:

(6c) $${\hat{E}}_{y}^{n+1}=k_{1}c_{1}{\hat{E}}_{y}^{n+1/2}+p_{4z}{\hat{F}}_{yz12}^{n+1/2}+p_{1z}{\hat{F}}_{yz1}^{n+1/2}+p_{2z}{\hat{F}}_{yz2}^{n+1/2}\;+p_{3z}\delta_{z}{\hat{H}}_{x}^{n+1/2}-p_{4x}{\hat{F}}_{yx12}^{n+1/2}-p_{1x}{\hat{F}}_{yx1}^{n+1/2}-p_{2x}{\hat{F}}_{yx2}^{n+1/2}-p_{3x}\delta_{x}{\hat{H}}_{z}^{n+1/2}$$

(6d) $${\hat{H}}_{z}^{n+1}+p_{3y}\delta_{y}{\hat{E}}_{x}^{n+1}=c_{1}{\hat{H}}_{z}^{n+1/2}+p_{4x}{\hat{G}}_{zx12}^{n+1/2}+p_{1x}{\hat{G}}_{zx1}^{n+1/2}\;+p_{2x}{\hat{G}}_{zx2}^{n+1/2}-p_{3x}\delta_{x}{\hat{E}}_{y}^{n+1/2}-p_{4y}{\hat{G}}_{zy12}^{n+1/2}-p_{1y}{\hat{G}}_{zy1}^{n+1/2}-p_{2y}{\hat{G}}_{zy2}^{n+1/2}$$

where the coefficients can be given as follows:

$k_{1}=\varepsilon_{0}/\left(\varepsilon_{0}+\sigma \Delta t\right)$, $c_{1}=\left(2-j\varpi \Delta t\right)/\left(2+j\varpi \Delta t\right)$, $c_{2}=2\Delta t/\left(2+j\varpi \Delta t\right)$, $c_{3}=\kappa_{\eta 1}^{-1}\kappa_{\eta 2}^{-1}+a_{\eta 2}\kappa_{\eta 1}^{-1}+a_{\eta 1}\kappa_{\eta 2}^{-1}+a_{\eta 1}a_{\eta 2}$, $a_{\eta n}=\left[\sigma_{\eta n}\exp\left(-v_{\eta n}\Delta t-1\right)\right]/\left(\sigma_{\eta n}\kappa_{\eta n}+\alpha_{\eta n}\kappa_{\eta n}^{2}\right)$, $b_{\eta n}=-v_{\eta n}\Delta t$, $p_{1\eta }=c_{2}k_{1}c_{3}\kappa_{z2}^{-1}$, $p_{2\eta }=c_{2}k_{1}c_{3}\kappa_{z2}^{-1}$, $p_{3\eta }=c_{2}k_{1}c_{3}\kappa_{z1}^{-1}\kappa_{z2}^{-1}/\Delta \eta$ and $p_{4\eta }=c_{2}k_{1}c_{3}$.

According to the leapfrog HIE procedure, the first time step can be given by substituting the magnetic components into electric components, one obtains:

(7) $$\begin{array}{l} \left(1-p_{3x}^{2}\delta_{2x}\right){\hat{E}}_{y}^{n+1/2}=\left(k_{1}c_{1}-p_{3z}^{2}\delta_{2z}\right){\hat{E}}_{y}^{n}\\ +p_{4z}{\hat{F}}_{yz12}^{n}+p_{1z}{\hat{F}}_{yz1}^{n}+p_{2z}{\hat{F}}_{yz2}^{n}-p_{4x}{\hat{F}}_{yx12}^{n}-p_{1x}{\hat{F}}_{yx1}^{n}-p_{2x}{\hat{F}}_{yx2}^{n}\\ +p_{1x}p_{3x}\delta_{x}{\hat{G}}_{zx1}^{n}+p_{2x}p_{3x}\delta_{x}{\hat{G}}_{zx2}^{n}-p_{4y}p_{3x}\delta_{x}{\hat{G}}_{zy12}^{n}-p_{1y}p_{3x}\delta_{x}{\hat{G}}_{zy1}^{n}-p_{2y}p_{3x}\delta_{x}{\hat{G}}_{zy2}^{n}\\ -p_{1z}p_{3z}\delta_{z}{\hat{G}}_{xz1}^{n}-p_{2z}p_{3z}\delta_{z}{\hat{G}}_{xz2}^{n}+p_{4y}p_{3z}\delta_{z}{\hat{G}}_{xy12}^{n}+p_{1y}p_{3z}\delta_{z}{\hat{G}}_{xy1}^{n}+p_{2y}p_{3z}\delta_{z}{\hat{G}}_{xy2}^{n}\\ +c_{1}p_{3x}\delta_{x}{\hat{H}}_{z}^{n}+p_{4x}p_{3x}\delta_{x}{\hat{G}}_{zx12}^{n}-p_{3y}p_{3x}\delta_{x}\delta_{y}{\hat{E}}_{x}^{n+1/2} \end{array}\;-c_{1}p_{3z}\delta_{z}{\hat{H}}_{x}^{n}-p_{4z}p_{3z}\delta_{z}{\hat{G}}_{xz12}^{n}-p_{3y}p_{3z}\delta_{z}\delta_{y}{\hat{E}}_{z}^{n+1/2}$$

By rewriting the second time step to the previous step of *n* − 1, substituting magnetic components to electric components accordingly, one obtains:

(8) $$\begin{array}{l} \left(1-p_{3z}^{2}\delta_{2z}\right){\hat{E}}_{y}^{n}=\left(k_{1}c_{1}+p_{3x}^{2}\delta_{2x}\right){\hat{E}}_{y}^{n-1/2}\\ +p_{4z}{\hat{F}}_{yz12}^{n-1/2}+p_{1z}{\hat{F}}_{yz1}^{n-1/2}+p_{2z}{\hat{F}}_{yz2}^{n-1/2}-p_{4x}{\hat{F}}_{yx12}^{n-1/2}-p_{1x}{\hat{F}}_{yx1}^{n-1/2}-p_{2x}{\hat{F}}_{yx2}^{n-1/2}\\ +p_{1x}p_{3x}\delta_{x}{\hat{G}}_{zx1}^{n-1/2}+p_{2x}p_{3x}\delta_{x}{\hat{G}}_{zx2}^{n-1/2}-p_{4y}p_{3x}\delta_{x}{\hat{G}}_{zy12}^{n-1/2}-p_{1y}p_{3x}\delta_{x}{\hat{G}}_{zy1}^{n-1/2}-p_{2y}p_{3x}\delta_{x}{\hat{G}}_{zy2}^{n-1/2}\\ +p_{1x}p_{3z}\delta_{z}{\hat{G}}_{zx1}^{n-1/2}+p_{2x}p_{3z}\delta_{z}{\hat{G}}_{zx2}^{n-1/2}-p_{4y}p_{3z}\delta_{z}{\hat{G}}_{zy12}^{n-1/2}-p_{1y}p_{3z}\delta_{z}{\hat{G}}_{zy1}^{n-1/2}-p_{2y}p_{3z}\delta_{z}{\hat{G}}_{zy2}^{n-1/2}\\ +p_{4x}p_{3x}\delta_{x}{\hat{G}}_{zx12}^{n-1/2}-c_{1}p_{3z}\delta_{z}{\hat{H}}_{x}^{n}-p_{3y}p_{3z}\delta_{z}\delta_{y}{\hat{E}}_{x}^{n-1/2} \end{array}\;+p_{4x}p_{3z}\delta_{z}{\hat{G}}_{zx12}^{n-1/2}+c_{1}p_{3x}\delta_{x}{\hat{H}}_{z}^{n}-p_{3y}p_{3z}\delta_{z}\delta_{y}{\hat{E}}_{z}^{n-1/2}$$

According to the leapfrog scheme, by adding Equations (7) and (8) together into a single equation, after some manipulations, one obtains:

(9) $$\begin{array}{l} \left(1-p_{3x}^{2}\delta_{2x}\right){\hat{E}}_{y}^{n+1/2}=\left(k_{1}^{2}c_{1}^{2}+k_{1}c_{1}p_{3x}^{2}\delta_{2x}\right){\hat{E}}_{y}^{n-1/2}+c_{1}p_{3x}\delta_{x}{\hat{H}}_{z}^{n}-c_{1}p_{3z}\delta_{z}{\hat{H}}_{x}^{n}\\ +\frac{k_{1}c_{1}p_{4z}}{2}\left({\hat{F}}_{yz12}^{n}+{\hat{F}}_{yz12}^{n-1}\right)+\frac{k_{1}c_{1}p_{1z}}{2}\left({\hat{F}}_{yz1}^{n}+{\hat{F}}_{yz1}^{n-1}\right)+\frac{k_{1}c_{1}p_{2z}}{2}\left({\hat{F}}_{yz2}^{n}+{\hat{F}}_{yz2}^{n-1}\right)\\ -\frac{k_{1}c_{1}p_{4x}}{2}\left({\hat{F}}_{yx12}^{n}+{\hat{F}}_{yx12}^{n-1}\right)-\frac{k_{1}c_{1}p_{1x}}{2}\left({\hat{F}}_{yx1}^{n}+{\hat{F}}_{yx1}^{n-1}\right)-\frac{k_{1}c_{1}p_{2x}}{2}\left({\hat{F}}_{yx2}^{n}+{\hat{F}}_{yx2}^{n-1}\right) \end{array}\;-k_{1}c_{1}p_{3y}p_{3z}\delta_{z}\delta_{y}\left({\hat{E}}_{x}^{n+1/2}+{\hat{E}}_{x}^{n-1/2}\right)-k_{1}c_{1}p_{3y}p_{3z}\delta_{z}\delta_{y}\left({\hat{E}}_{z}^{n+1/2}+{\hat{E}}_{z}^{n-1/2}\right)$$

The auxiliary variables can be given as, for example,

(10a) $${\hat{F}}_{x\eta 1}^{n+1/2}=a_{\eta 2}\sum_{m=0}^{n-1}e^{-v_{\eta 2}m\Delta t}\partial_{\eta }{\hat{H}}_{\tilde{\eta }}^{n-m}$$

(10b) $${\hat{F}}_{x\eta 2}^{n+1/2}=a_{\eta 1}\sum_{m=0}^{n-1}e^{-v_{\eta 1}m\Delta t}\partial_{\eta }{\hat{H}}_{\tilde{\eta }}^{n-m}$$

(10c) $${\hat{F}}_{x\eta 12}^{n+1/2}=a_{\eta 1}a_{\eta 2}\sum_{m=0}^{n-1}e^{-v_{\eta 1}m\Delta t}\sum_{m=0}^{n-1}e^{-v_{\eta 2}m\Delta t}\partial_{\eta }{\hat{H}}_{\tilde{\eta }}^{n-m}$$

where $\tilde{\eta }$ denotes the complement of η, for example, η=y, and $\tilde{\eta }=z$. In order to solve the summation term in Equation (10a–c), the recursive convolution (RC) method is employed during the calculation. According to the RC method, the results of auxiliary variables can be given as:

(11a) $${\hat{F}}_{x\eta 1}^{n+1/2}=b_{\eta 2}{\hat{F}}_{x\eta 1}^{n-1/2}+a_{\eta 2}\partial_{\eta }{\hat{H}}_{\tilde{\eta }}^{n}$$

(11b) $${\hat{F}}_{x\eta 2}^{n+1/2}=b_{\eta 1}{\hat{F}}_{x\eta 2}^{n-1/2}+a_{\eta 1}\partial_{\eta }{\hat{H}}_{\tilde{\eta }}^{n}$$

(11c) $${\hat{F}}_{x\eta 1}^{n+1/2}=b_{\eta 1}b_{\eta 2}{\hat{F}}_{x\eta 1}^{n-1/2}+a_{\eta 1}a_{\eta 2}\partial_{\eta }{\hat{H}}_{\tilde{\eta }}^{n+1/2}$$

According to the convexity of Maxwell’s equations, the other auxiliary variables and components can be solved by employing a similar approach. By employing the above-mentioned equations, leapfrog HIE procedure with CPML formulation and CE method can be implemented. The entire update procedure of the described is as follows:

(1) Explicitly update $E_{x}^{n+1/2}$, $E_{z}^{n+1/2}$, in the half-integer time step;
(2) Explicitly update $H_{x}^{n}$, $H_{z}^{n}$ in the integer time step;
(3) Implicitly update $E_{y}^{n+1/2}$ and $H_{y}^{n+1/2}$ along the directions of fine details;
(4) Explicitly update auxiliary variables inside PML regions.

**Figure 1:**
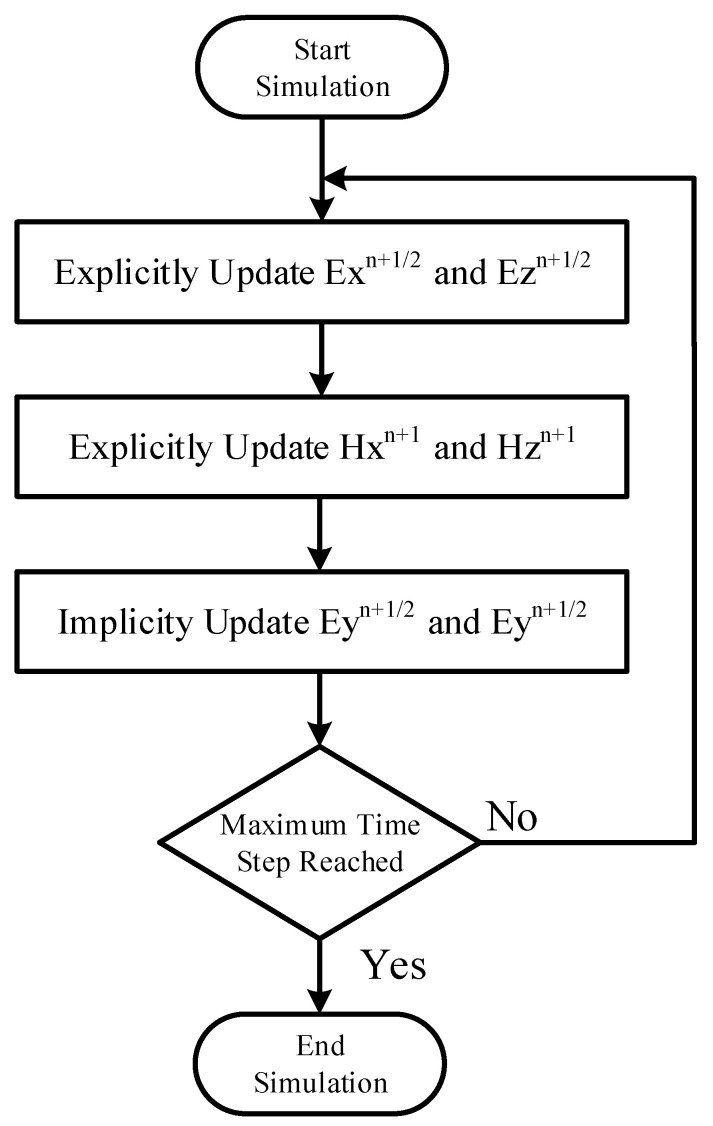
The block diagram of the entire update procedure.

The block diagram of the entire update procedure is shown in Figure 1 which corresponds to the above-mentioned procedure. In order to demonstrate the simulation duration and memory consumption of different PML algorithms, a number of operators are employed during comparison. During the algorithm implementation, a calculation of multiplication/division and addition/subtraction determine the efficiency and memory in theory. Multiplication/division operators and implicit equations occupy much more resources compared with addition/subtraction operators and explicit equations, respectively. Thus, it is important to analyze the influence of multiplication/division and addition/subtraction numbers on efficiency and resources. The compare different PML algorithms, FDTD-PML in [40], HIE-PML in [33], LHIE-CPML in [34], CE-HIE-HPML in [41], CE-LHIE-HPML and FDTD-HPML in [42] with explicit and implicit equations are employed with results that are shown in Table 1.

As can be concluded from Table 1, the explicit algorithms show the most considerable performance is due to the non-calculation of matrices. Due to the introduction of the implicit scheme, a number of multiplication/division and addition/subtraction operators show significant improvement, resulting in increments of simulation duration and consumption memory. Meanwhile, due to the employment of the higher order formulation, algorithms also show significant increments in efficiency and resources at the same time. To be more precise, the leapfrog scheme and CPML formulation show a decrement in computational complexity. Such conditions result in a memory and simulation duration decrement by CPML and leapfrog schemes compared to the CFS-PML and its original schemes, respectively. Due to the introduction of the CE method, several coefficients and operators should be calculated at each time step resulting in the increment of memory. In conclusion, the proposed algorithm can reduce the complexity of the algorithm compared with the other PML formulations based on the HIE procedure. However, in the bandpass simulation, the time step can be enlarged according to the percent bandwidth of the source. Owing to such conditions, the CE method can significantly improve the entire performance during the simulation.

## 3. Numerical Results and Experiments

The effectiveness of the proposed scheme can be demonstrated through employing the numerical example and experiment including a filter for the sensors system with experiments and remote sensing problems with theory resolution. The computer for the code holds the parameter of i7-11700k and DDR4 128 GB. The program is implemented by Fortran under Visual Studio. The entire computation duration can be described as the following procedure: (1) Import geometry structures with STL files; (2) Set condition for calculation including time step, mesh size and more; (3) Start Calculation; (4) Export results for TXT file.

### 3.1. Micro-Strip Filter for Sensors System

**Figure 2:**
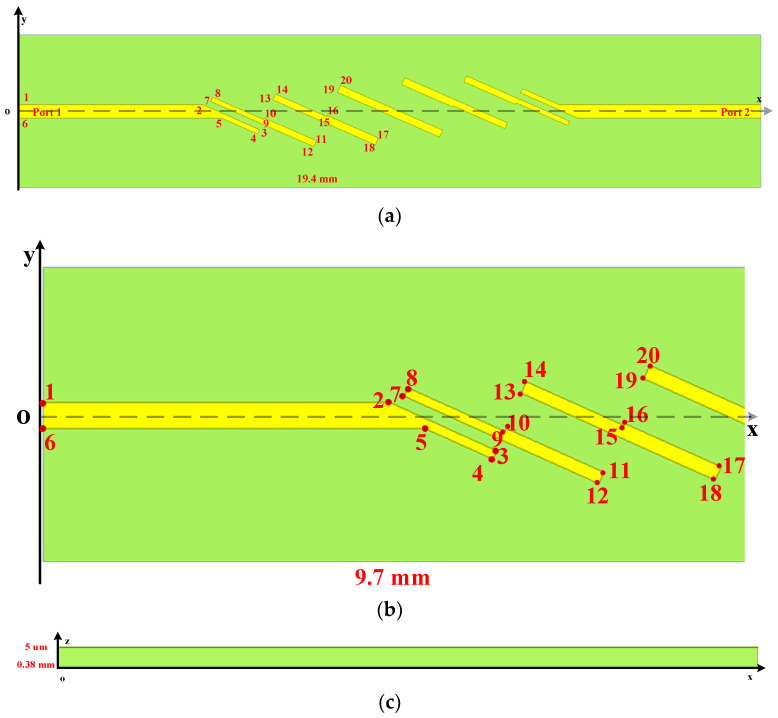
The sketch picture of the filter for sensors system: (**a**) top view; (**b**) top view enlargement; (**c**) front view.

Within microwave components, micro-strip components can be regarded one of the most powerful and widely spread. Among some of them, an extremely thin metal patch is located on the surface of the device. Thus, by employing the unconditionally stable algorithms and conventional explicit algorithms directly, uniform extremely fine mesh must be employed within the entire computational domain which results in significant increments of memory and duration. In order to alleviate such conditions, the HIE procedure is introduced for the fine details along one and two dimensions. Here, a micro-strip filter for sensors system is employed for the illustration. The sketch picture of the structure is shown as Figure 2. Through the establishment of a rectangular coordinate system, a complex and oblique patch can be expressed and located by the specific coordinate location, as shown in Table 2. Along the vertex coordinates of the patch, the entire structure can be established in the CAD mechanical drawing. The coordinate origin is located at the middle of the left bottom edge. In order to clarify the position of each point, Figure 2a shows each point of the patch from xoy plane. The enlargement of the left side of the structure is shown in Figure 2b. The right side of the structure can be obtained through the symmetry principle.

The entire filter structure is composed of substrate and patch. The dielectric substrate with the electrical parameter of $\varepsilon_{r}=9.4$ holds the dimensions of 19.4×4×0.38 mm. The gold patches whose parameters can be founded in [43] are located on the surface of the devices. The entire model can be regarded as the center–symmetry model. The excitation port 1 and port 2 with the width and length of 0.34 mm and 0.385 mm are located at the left and bottom of the devices, respectively. The height of substrate and patch along the z-direction are 0.38 mm and 5 mm, respectively. It can be observed from Figure 2c that the entire structure holds the fine details along z-direction. By applying uniform mesh sizes directly to the entire structure, large computational domains will occur resulting in an unacceptable simulation.

**Figure 3:**
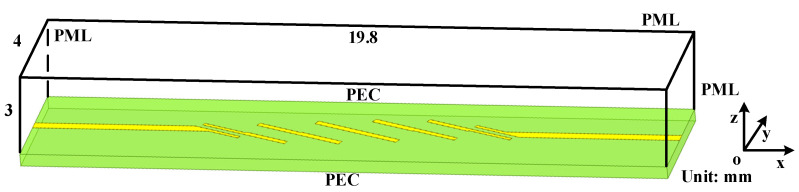
The sketch picture of the entire filter computational domain.

The entire computational domain is shown in Figure 3. As can be observed, the entire computational domain holds the parameters of 19.8×4×3.385 mm. At the boundaries of x-directions and the top boundary of z-direction, the 10-cell-PML is employed for termination to absorb outgoing waves. The other boundaries are terminated by the perfectly electronic conductor (PEC). The source with the center frequency and bandwidth of 20 GHz and 5 GHz propagates at port 1, along the positive side along x-direction which corresponds to the percent bandwidth of %B=25. The observation point is located at the corner of port 2 for observing the wave reflection. In order to obtain the best absorption, both in the time domain and frequency domain, the parameters inside the PML regions are chosen to achieve such conditions. The parameters of the higher order PML algorithms are $\kappa_{\eta 1}=12$, $\alpha_{\eta 1}=1.1$, $m_{\eta 1}=3$, $\sigma_{\eta 1\max}=2.6\sigma_{\eta 1opt}$, $\kappa_{\eta 2}=11$, $\alpha_{\eta 2}=0.001$, $m_{\eta 2}=4$ and $\sigma_{\eta 2\max}=3.0\sigma_{\eta 2opt}$, where

(12) $$\sigma_{\eta nopt}=\left(m_{\eta n}+1\right)/\left(150\cdot \pi \cdot \Delta \right)$$

**Figure 4:**
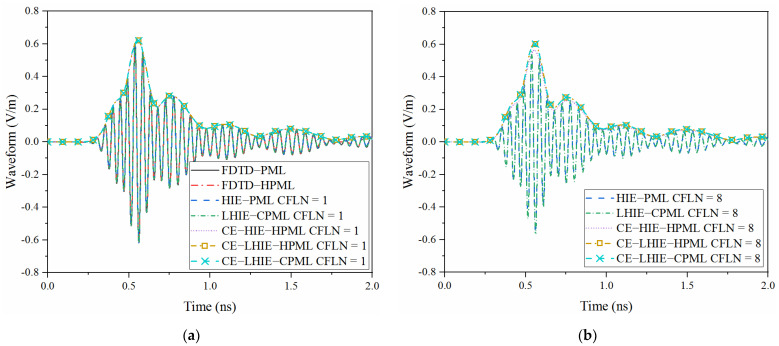
Waveform obtained by different PML algorithms at the observation point: (**a**) CFLN = 1; (**b**) CFLN = 8.

For comparison, parameters of CFS-PML regions are $\kappa_{\eta }=6$, $\alpha_{\eta }=1.7$, $m_{\eta }=2$ and $\sigma_{\eta \max}=3.2\sigma_{\eta opt}$. In order to maintain the accuracy of calculation, non-uniform mesh sizes are employed in the HIE algorithm with the parameters of Δx=Δy=0.2 mm and Δz=0.0025 mm. The uniform mesh sizes of the conventional FDTD algorithm are chosen as Δx=Δy=Δz=Δ=0.0025 mm. Thus, time step in the explicit and implicit algorithms are chosen as 0.48 fs and 4.32 fs, respectively. It can be concluded that the CE method can significantly enlarge the time step in the bandpass simulation. The accuracy of the algorithm in the time domain can be evaluated by the waveform obtained by a different PML algorithm in the time domain at the observation point, shown as Figure 4.

Waveforms obtained by explicit algorithms and implicit algorithms with CFLN = 1 are almost overlapped, as shown in Figure 4a. Due to the employment of bandpass-sampling-theorem, the complex envelope method generates the envelope of waveform. Meanwhile, waveforms obtained by the CE method also overlapped with the boundaries of the waveform obtained by the explicit algorithms. Such conditions indicate that these algorithms almost hold the same accuracy. The waveforms obtained by different PML algorithms with large CFLNs are shown in Figure 4b. Compared with the waveform with CFLN = 1, waveforms show significant shifting which indicates the degeneration of calculation accuracy. Compared with the lowpass-sampling-theorem based algorithms, bandpass-sampling-theorem based algorithms show less curves shifting which indicates a better computational accuracy with the circumstance of larger CFLNs. The curve obtained by the proposed scheme is overlapped with that obtained by the CE-LHIE-CPML scheme. Such conditions indicates that these algorithms hold the same accuracy. Meanwhile, it shows the least shifting compared to the other implicit algorithms with larger CFLNs. Such conditions indicates that the proposed algorithm has the best accuracy among these implicit algorithms.

**Figure 5:**
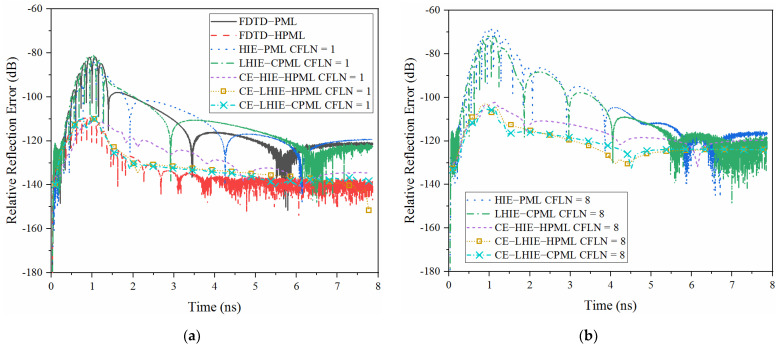
The relative reflection error obtained by different PML algorithms in the time domain: (**a**) CFLN = 1; (**b**) CFLN = 8.

The wave reflection at the boundaries of the domain also affects the accuracy of the calculation. The wave reflection can be reflected by the relative reflection error in the time domain which can be defined as:

(13) $$R_{dB}\left(t\right)=20\log_{10}\left[\left|E_{t}^{}\left(t\right)-E_{r}^{}\left(t\right)\right|/\left|\max\left\{E_{r}^{}\left(t\right)\right\}\right|\right]$$

where $E_{t}^{}\left(t\right)$ is the test solution which can be directly obtained at the observation point and $E_{r}$ is the reference solution. The reference solution can be calculated by the 20 times enlarged computational domain with 128-cell-PML regions. By employing such a circumstance, reflection wave at the boundaries of the domain can be ignored during the evaluation. Figure 5 shows the relative reflection error in the time domain obtained by different PML algorithms.

The relative reflection error obtained by different PML algorithms with CFLN = 1 is shown in Figure 5a. It can be concluded that the absorption decreases slightly compared with the conventional explicit algorithms due to the introduction of the matrix calculation. Thus, LHIE-PML and LHIE-CPML schemes almost hold the same performance which is inferior compared to the FDTD-PML. The performance can be significantly improved by employing the higher order formulation from both the aspect of late-time reflections and maximum relative reflection error (MRRE). In addition, by employing the CE method, absorption can be significantly improved in the bandpass circumstance. Thus, bandpass-sampling-theorem based algorithms can obtain better performance compared with the FDTD-HPML. It can be concluded that the CE-LHIE-HPML and CE-LHIE-CPML algorithms can obtain better performance compared to the CE-HIE-PML. The reason is that the LHIE algorithm can obtain better numerical dispersion compared to the original HIE algorithm, resulting in such a condition. Figure 5b shows the relative reflection error in the time domain obtained by different PML algorithms with larger CFLNs. The absorption decreases with the enlargement of CFLNs due to the increment of numerical dispersion with the enlargement of time steps. It can also be concluded that LHIE algorithm can obtain better performance compared with the HIE algorithm, which can be reflected by the LHIE-CPML, CE-LHIE-HPML and CE-LHIE-CPML algorithms compared with the HIE-PML and CE-HIE-HPML, respectively. The CE-LHIE-HPML can obtain almost the same, compared with the proposed CE-LHIE-CPML. Most importantly, performance shows its significant advantages with larger CFLNs, compared to the FDTD-PML in bandpass circumstance.

It can be concluded from Table 3 that the employment of higher order formulation and CE method results in the increment of simulation duration and computational resources due to the increment of many more coefficients, auxiliary variables, field components and matrices. Thus, compared with the PML scheme and explicit schemes, time and memory occupied by the HPML and HIE procedure increases significantly. Although the CE method slightly increases the memory consumption during the simulation, iteration steps decrease at the same time due to the enlargement of the time step. It should be noticed that CE method alleviates the time increment problem of the implicit algorithm with lower CFLNs. The proposed scheme can obtain a 13.7% time improvement compared to the FDTD-PML. With the improvement of CFLNs, simulation duration can be decreased by enlarging the time step resulting in an improvement of efficiency. With CFLN = 8, the CE method based implicit algorithm shows more than an 80% time improvement compared with the FDTD-PML. In summary, through the time domain simulation, the proposed scheme can obtain the same accuracy and absorption compared with the previous work which is based on the bandpass-sampling-theorem. Meanwhile, the memory occupation and simulation duration can be decreased significantly.

**Figure 6:**
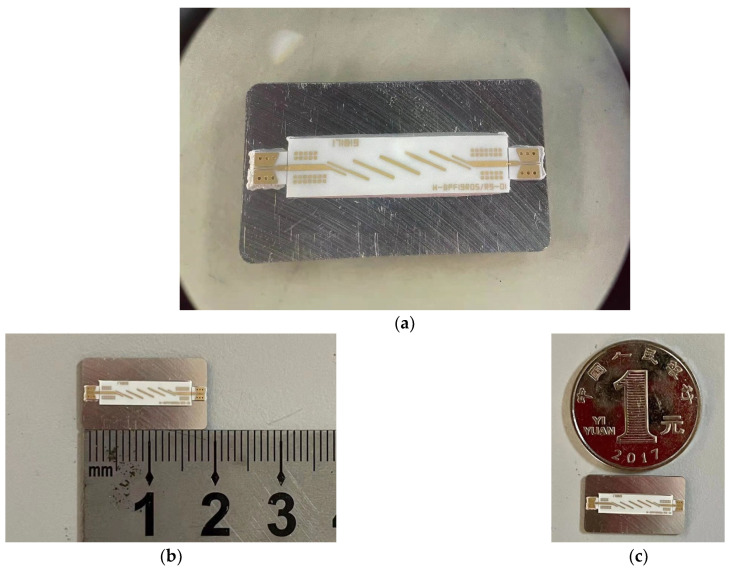
The picture of: (**a**) entire filter model; (**b**) detail size of the filter with ruler; (**c**) detail size with coin with the radius of 25 mm.

The above-mentioned discussion verifies the effectiveness of the algorithm in the time domain. In order to further testify the algorithm in the frequency domain, a filter for the sensors system is simulated and manufactured. The scattering parameter is one of the most important parameters in the evaluation. Here, return loss (S_11_) and transmission loss (S_21_) are considered in the frequency domain. The photograph of the filter and its sketch size is shown in Figure 6 which is compared with the coin with radius of 25 mm.

**Figure 7:**
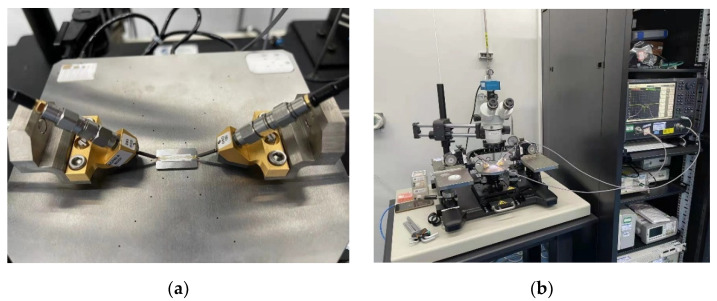
The picture of: (**a**) probe operation table; (**b**) measured system.

In order to demonstrate the performance of the filter in the frequency domain, results are measured through the following system, shown as Figure 7. Due to the fact the excitation ports hold extremely fine details, the excitation source with a probe is employed to excite the filter structure. The probe at each port is also employed to collect the reflection wave and transmission wave. At the other end of the cable, a spectrum analyzer is employed for the evaluation of performance. The return loss and transmission loss can be measured from such a system.

**Figure 8:**
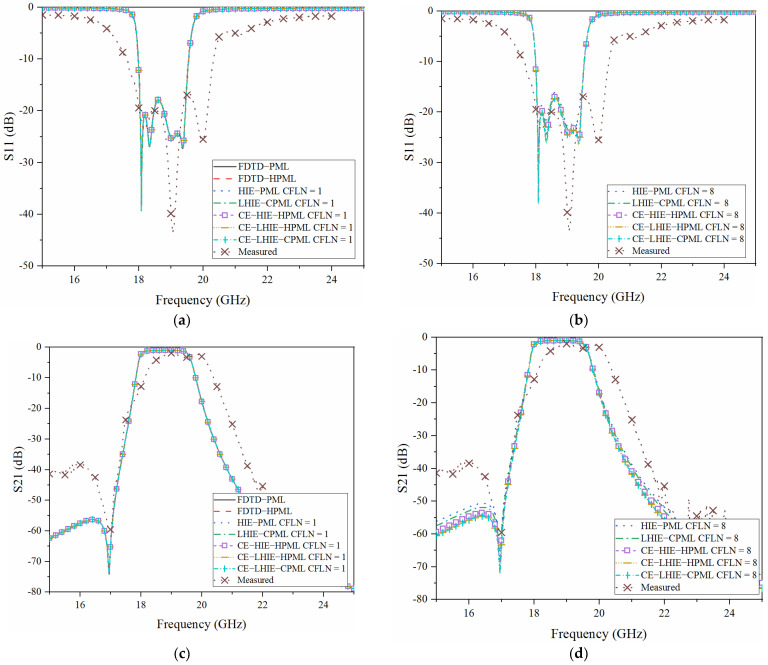
The scattering parameter obtained by different PML algorithms: (**a**) SCFLN = 1; 1with CFLN = 1; (**b**) S11 with CFLN = 8; (**c**) S21 with CFLN = 1; (**d**) S21 with CFLN = 8.

Figure 8 shows S_11_ and S_21_ obtained by different PML algorithms and its experiments, respectively. Through Figure 8a,c, it can be observed that these curves obtained by different PML algorithms are almost overlapped. Such conditions indicate that these algorithms almost hold the same computational accuracy during the calculation in the entire frequency band. Through Figure 8b,d, it can be observed that the curves show shifting compared to the lower CFLNs. Such conditions indicate that the accuracy of algorithms decrease with the enlargement of CFLNs. The reason is that the numerical dispersion increases with the enlargement of the time step which results in the degeneration of accuracy. The curves obtained by the proposed scheme and CE-LHIE-HPML hold the least shifting, indicating the best accuracy. It can also be concluded that compared with the lowpass-sampling-theorem based algorithms, algorithms that are based on the CE method hold better accuracy in the entire frequency band. In summary, simulation and measurement shows the same conclusion with that obtained in the time domain simulation. In addition, all of these algorithms show considerable agreement with the experiment. Such a condition indicate that the proposed algorithm can be extensively employed in practical engineering.

However, there is still error between simulation and experiment. The reason can be described from the following aspects: (1) The manufacturing process tolerance results in disagreement compared to the size in the simulation. The generation of shifting is caused by such a condition. Thus, the manufacturing process tolerance overcoming the technique becomes increasingly important; (2) The measurement of the filter system is a precision-type instrument, especially for the probe. The pollution of the devices also leads to shifting in the experiment; (3) Due to the existence of the complex and oblique patch, the conventional conformal method in the Yee’s grid becomes inaccurate. Conformal error causes occur at the oblique and complex part. Such conditions also result in the curves shifting. Thus, improvement on the conformal method for the HIE algorithm is also worth investigating.

### 3.2. Remote Sensing Problem with Theory Resolution

**Figure 9:**
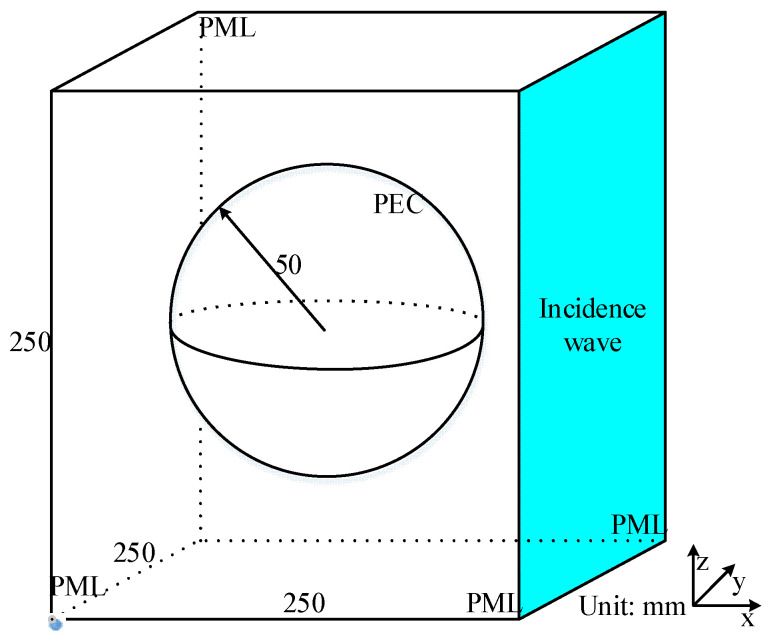
The sketch picture of the metal sphere model inside the entire computational domain.

The performance of the proposed algorithm has been demonstrated by employing the above-mentioned numerical example and its corresponding experiment. Through results, its behaviors show considerable performance during the whole simulation. In order to further demonstrate the efficiency in much more complex problems, a metal sphere model is considered. The normal vector of the sphere model ranges from −180 degree to 180 degree which holds the most complexity among all of the structures. Because the metal sphere model in the sensing problem holds theory resolution, calculation accuracy can be demonstrated through the comparison. Figure 9 demonstrates the sketch picture of the remote sensing problem with the metal sphere.

The entire computational domain holds dimensions of 250×250×250 in each direction. The metal sphere model with the radius of 50 mm can be regarded as the PEC, located at the center of the domain. The modulated Gaussian pulse with the center frequency and maximum frequency of 1.5 GHz and 2.0 GHz propagates along the negative side along x-direction, whose percent bandwidth can be obtained as %B=50. At the boundaries of the domain, all of the boundaries are terminated by 10-cell-PML for wave reflection. The parameters of higher order PML algorithms are $\kappa_{\eta 1}=100$, $\alpha_{\eta 1}=2.5$, $m_{\eta 1}=2$, $\sigma_{\eta 1\max}=0.5\sigma_{\eta 1opt}$, $\kappa_{\eta 2}=3$, $\alpha_{\eta 2}=1.3$, $m_{\eta 2}=1$ and $\sigma_{\eta 2\max}=0.01\sigma_{\eta 2opt}$. For comparison, parameters of CFS-PML regions are $\kappa_{\eta }=170$, $\alpha_{\eta }=2.1$, $m_{\eta }=3$ and $\sigma_{\eta \max}=0.8\sigma_{\eta opt}$. In order to make a comparison of computational accuracy between different algorithms, uniform mesh size is employed as Δx=Δy=Δz=Δ=2.5 mm. Thus, the entire computational domain can be discretized as 100Δx×100Δy×100Δz. The time step of the lowpass-sampling-theorem based and bandpass-sampling-theorem based algorithms are 14.4 fs and 57.6 fs, respectively.

**Figure 10:**
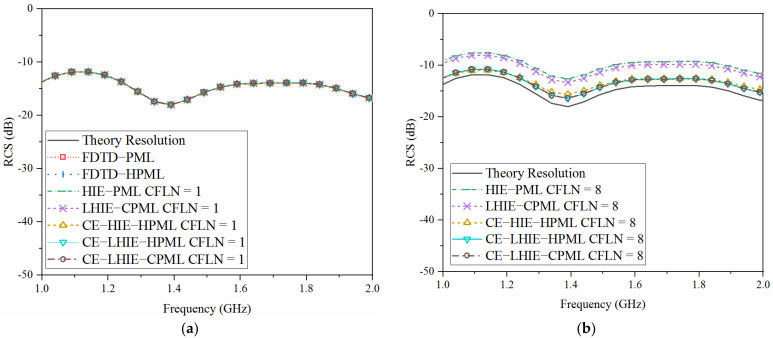
Radar cross section obtained by different PML algorithms: (**a**) CFLN = 1; (**b**) CFLN = 8.

The accuracy of the algorithm can be reflected by the radar cross section (RCS) parameter in the frequency domain. Figure 10 shows the RCS obtained by different PML algorithms and CFLNs in the frequency domain and its theory resolution. As can be obtained from Figure 10a, all of the curves are overlapped. Such conditions indicate that all of the algorithms hold the same accuracy with CFLN = 1. However, the curves show shifting compared to the theory resolution. The reason is that numerical dispersion and reflection waves increase at the same time with the enlargement of CFLNs. It can be concluded that the proposed algorithm shows the least shifting. Such conditions indicate that it holds the best accuracy in the frequency domain with larger CFLNs. The effectiveness of the algorithm can also be reflected by the computational duration, consumption memory, iteration step, memory increment and time reduction obtained by different PML algorithms, as shown in Table 4.

It can be concluded from Table 4 that the bandpass-sampling-theorem can significantly improve the calculation efficiency due to the enlargement of time steps and decrement of iteration steps. Compared with the original HIE procedure, the one-step leapfrog scheme can update the components at the half-integer and integer steps, respectively. Such conditions also improve the performance of the entire algorithm. In summary, it can obtain the same conclusion as the previous example, that the proposed scheme holds the best accuracy, efficiency and resources compared to the existed algorithms.

Due to the existence of a massive absorbing boundary condition, the choice of absorbing boundary condition according to different simulation problems has raised much attention. There are several widely-spread absorbing boundary conditions including perfectly matched layer and transparent boundary conditions (TBC) [44]. It can be concluded from the simulation that higher order PML formulation can significantly enhance the absorption at low-frequency band due to the absorption of the low-frequency propagation waves. The employment of higher order PML formulation is available for problems with large amounts of low-frequency waves. For the simulation without much low-frequency waves, the unsplit CFS-PML formulation shows its advantages in late-time reflections and low-frequency evanescent waves. For the higher frequency range applications, due to the non-absorption of plasmonic waves in optical waveguide devices, TBC formulation shows its significant advantages with great potential.

## 4. Conclusions

In order to efficiently simulate devices for a sensors system in narrow bandpass circumstances, the one-step leapfrog HIE algorithm with the complex envelope method and higher order CPML formulation is proposed. Through the implementation, dielectric material with fine details along one and two dimensions can be solved. The numerical example and its experiment are carried out for the demonstration of effectiveness. It can be concluded from the results that the proposed algorithm shows its advantages in accuracy, efficiency, absorption and computational resources, both in the time domain and frequency domain. Meanwhile, the CFL condition can be broken in the proposed scheme which also indicates the efficiency improvement in the entire simulation. Compared with the previous work, it can maintain considerable performance with decrement of computational resources and simulation duration.

In future work, the method to reduce memory consumption and simulation duration increment caused by matrix solution in the HIE procedure can be further investigated. Meanwhile, numerical dispersion increases with the enlargement of the time step, which affects the calculation accuracy significantly. The method to decrease numerical dispersion with a larger time step is worth investigating. Finally, conformal errors still exist in the complex and oblique structures. Improvements on the conformal method for the HIE algorithm is also worth investigating.

## Figures and Tables

**Table 1:** The multiplication/division and addition/subtraction operators with explicit and implicit equations in different algorithms.

PML Algorithm	Addition/Subtraction		Multiplication/Division		Total Operators
	Implicit	Explicit	Implicit	Explicit	
FDTD-PML	0	60	0	42	102
FDTD-HPML	0	90	0	78	168
HIE-PML	40	54	36	28	158
LHIE-CPML	36	54	32	28	150
CE-HIE-HPML	66	72	54	36	228
CE-LHIE-HPML	54	72	36	36	198
Proposed	48	66	40	36	190

**Table 2:** The specific coordinate location of each point in Figure 2a,b on the surface of the model in (x, y, z) form (unit: mm).

Point in Figure 2a,b	Specific Coordinate Location (x, y, z)	Point in Figure 2a,b	Specific Coordinate Location (x, y, z)
1	(−9.7, −0.175, 0.005)	2	(−5.3, −0.175, 0.005)
3	(−3.4, 0.48, 0.005)	4	(−3.46, 0.59, 0.005)
5	(−4.4, 0.175, 0.005)	6	(−9.7, 0.175, 0.005)
7	(−4.7, −0.25, 0.005)	8	(−4.64, −0.25, 0.005)
9	(−3.3, 0.22, 0.005)	10	(−3.27, 0.18, 0.005)
11	(−1.9, 0.77, 0.005)	12	(−2.0, 0.93, 0.005)
13	(−3.1, −0.3, 0.005)	14	(−3, −0.45, 0.005)
15	(−1.64, 0.11, 0.005)	16	(−1.64, 0.11, 0.005)
17	(−3.1, 0.69, 0.005)	18	(−0.4, 0.88, 0.005)
19	(−3.07, −0.29, 0.005)	20	(−3, −0.45, 0.005)

**Table 3:** The computational duration, consumption memory, iteration step, memory increment and time reduction obtained by different PML algorithms.

PML Algorithm	CFLN	Steps	Memory (GB)	Memory Increment (%)	Time (min)	Time Reduction (%)
FDTD-PML	1	65,536	0.5	-	21.9	-
FDTD-HPML	1	65,536	0.9	80	38.4	−75.3
HIE-PML	1	65,536	0.9	80	41.7	−90.4
LHIE-CPML	1	65,536	0.8	60	38.3	−42.8
CE-HIE-HPML	1	7282	1.4	180	24.0	−9.5
CE-LHIE-HPML	1	7282	1.3	160	22.1	−9.0
CE-LHIE-CPML	1	7282	1.1	120	18.9	13.7
HIE-PML	8	8192	0.9	80	12.6	42.5
LHIE-CPML	8	8192	0.8	60	10.3	53.0
CE-HIE-HPML	8	911	1.4	180	4.6	79.0
CE-LHIE-HPML	8	911	1.3	160	3.5	84.0
CE-LHIE-CPML	8	911	1.1	120	2.9	86.8

**Table 4:** The computational duration, consumption memory, iteration step, memory increment and time reduction obtained by different PML algorithms in the sphere model.

PML Algorithm	CFLN	Steps	Memory (GB)	Memory Increment (%)	Time (min)	Time Reduction (%)
FDTD-PML	1	65,536	0.4	-	4.6	-
FDTD-HPML	1	65,536	0.8	−100	10.2	−152.4
HIE-PML	1	65,536	0.8	−100	13.7	−197.8
LHIE-CPML	1	65,536	0.7	−75	10.9	−137.0
CE-HIE-HPML	1	7282	1.1	−175	4.9	−6.5
CE-LHIE-HPML	1	7282	1.0	−150	4.7	−2.2
CE-LHIE-CPML	1	7282	0.8	−100	4.4	4.3
HIE-PML	8	8192	0.8	−100	2.7	45.7
LHIE-CPML	8	8192	0.7	−75	2.3	50.0
CE-HIE-HPML	8	911	1.1	−175	0.7	84.8
CE-LHIE-HPML	8	911	1.0	−150	0.6	87.0
CE-LHIE-CPML	8	911	0.8	−100	0.4	91.3

## Data Availability

The data presented in this study are available on request from the corresponding author.
